# Severe Coronary Artery Ectasia in a 31-Year-Old Presenting With an Inferior ST-Elevation Myocardial Infarction: A Case Report

**DOI:** 10.7759/cureus.24340

**Published:** 2022-04-21

**Authors:** Nicholas Venuti, Andrew Mangano

**Affiliations:** 1 Internal Medicine, Grand Strand Medical Center, Myrtle Beach, USA

**Keywords:** thrombus, triple therapy, dual antiplatelet therapy, ectatic coronary vessels thrombus, coronary artery ectasia

## Abstract

Coronary artery ectasia (CAE) is a rare dilation of the lumen in coronary arteries, either localized to one vessel or diffuse in multiple vessels. A 31-year-old white male with no significant past medical or cardiac history, presented with severe sudden onset chest pain, diaphoresis, shortness of breath, and nausea without vomiting. A 12 lead electrocardiogram (EKG) showed an ST elevation in the inferior leads with reciprocal changes, suggestive of myocardial infarction in the right coronary artery (RCA). He was taken directly to the catheterization laboratory for coronary angiography, which identified a mid-RCA thrombus with thrombolysis in myocardial infarction (TIMI)-1 flow, and distal to that in the right posterolateral branch was another thrombus with TIMI-0 flow. Additionally, he was found to have severely diffuse CAE in all of his coronary arteries. Angioplasty successfully restored TIMI-3 flow throughout the RCA. We present this case to discuss the prevention of complications from CAE. There are currently no recommendations on the use of antiplatelet or anticoagulation therapy in patients with CAE.

## Introduction

Coronary artery ectasia (CAE) is a rare dilation of the lumen in coronary arteries, either localized to one vessel or diffuse in multiple vessels. In one study of 20,000 left heart catheterizations, CAE had a prevalence of only 0.85%, with a male predominance of more than 90% [1]. Additionally, it is suggested that only 23.7% have isolated CAE, while the remaining also has significant coronary artery disease (CAD) [2]. CAE dilation is defined as a diameter 1.5 times larger than an adjacent normal coronary artery and must include at least one-third of the affected artery's length. CAE differs from coronary aneurysms, which are identified by a focal dilation. Several studies have suggested that the most commonly involved coronary artery is the right coronary artery (RCA) [1,2,3]. There are four classifications of CAE: Type 1 - diffuse ectasia in two or more vessels; Type 2 - diffuse ectasia in one vessel, and localized ectasia in another; Type 3 - diffuse ectasia limited to one vessel; Type 4 - localized ectasia limited to one vessel [4].

The pathophysiology of CAE is poorly understood; however, it could be considered as excessive remodeling by proteolytic enzyme degradation of the extracellular matrix and overexpression of matrix metalloproteinases resulting in luminal expansion [5]. Some have suggested possible etiologies to include: Kawasaki disease, atherosclerosis, mycotic or septic emboli, Marfan syndrome, Takayasu disease, systemic lupus erythematosus, polyarteritis nodosa, or Loeys-Dietz syndrome. An overwhelming majority of CAE is incidentally detected during coronary angiography or computed tomography (CT). However, a thrombus in the dilated artery may embolize distally to a smaller segment of the vessel, resulting in a presentation with acute myocardial infarction (AMI) [6].

Because CAE is so rare, it has been challenging for the research community to conduct large trials on how to appropriately manage these patients. As a result, there has not been a consensus on preventative treatment to include an antiplatelet and/or an anticoagulant, versus dual antiplatelet therapy (DAPT), versus triple therapy (DAPT plus anticoagulation). Additionally, if preventative treatment is advised there is no recommendation on whether it should be for a short duration or indefinitely.

## Case presentation

**Figure 1:**
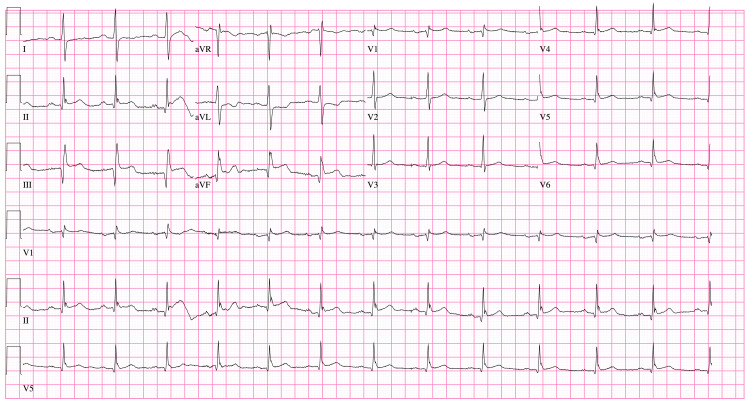
Initial emergency room electrocardiogram Initial EKG showed a regular rate at 78 beats per minute, narrow QRS, regular rhythm, and ST elevations in leads II, III, aVF.

A 31-year-old white male with no significant past medical or cardiac history presented to the emergency department (ED) via ambulance with severe sudden onset chest pain, which started about one hour prior to his arrival. His chest pain was associated with diaphoresis, shortness of breath, and nausea without vomiting. The patient reported that his father died at the age of 35 from an AMI, and his father’s mother also died prior to the age of 40 from an AMI. In the ED, a 12 lead electrocardiogram (EKG) showed inferior ST elevations with reciprocal ST depressions (Figure 1), and a mild troponin of 0.018. He was taken directly to the cath lab for further management, where his chest pain continued.

**Figure 2:**
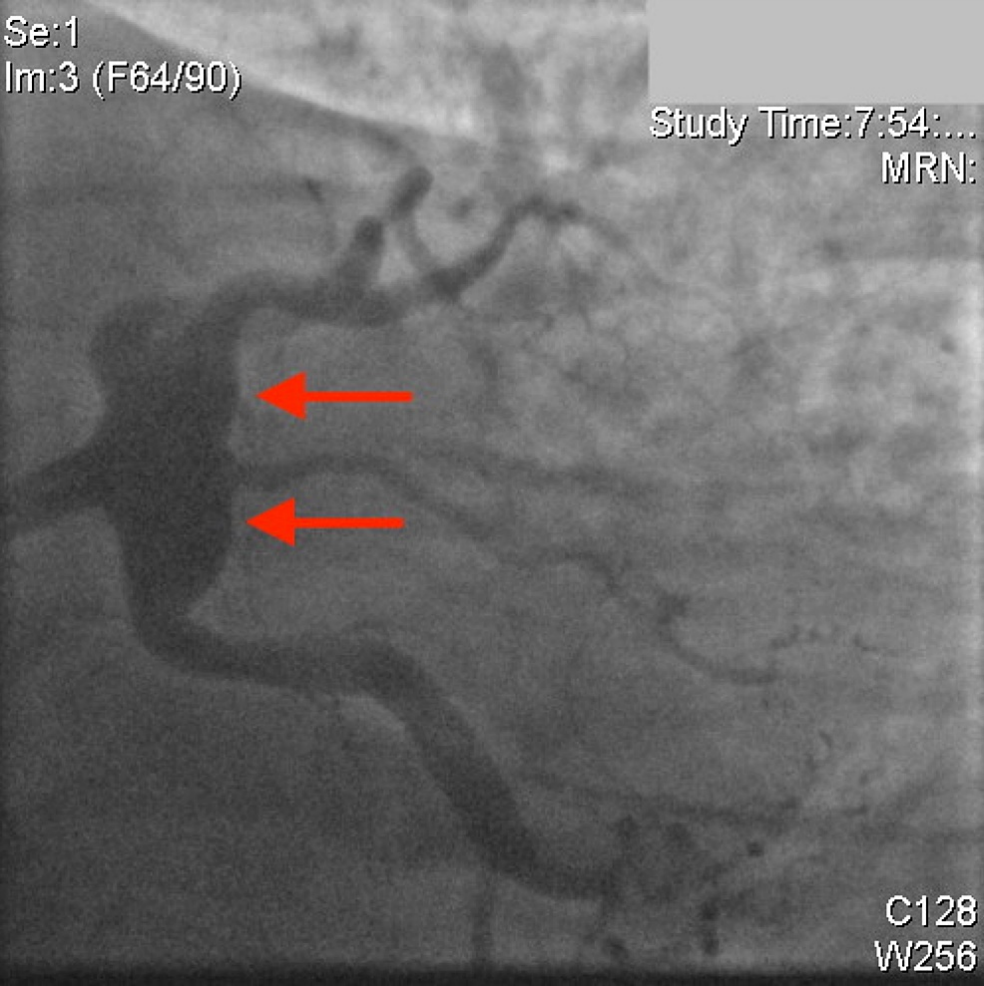
Left anterior descending artery, left circumflex artery, left main artery, ramus intermedius

**Figure 3:**
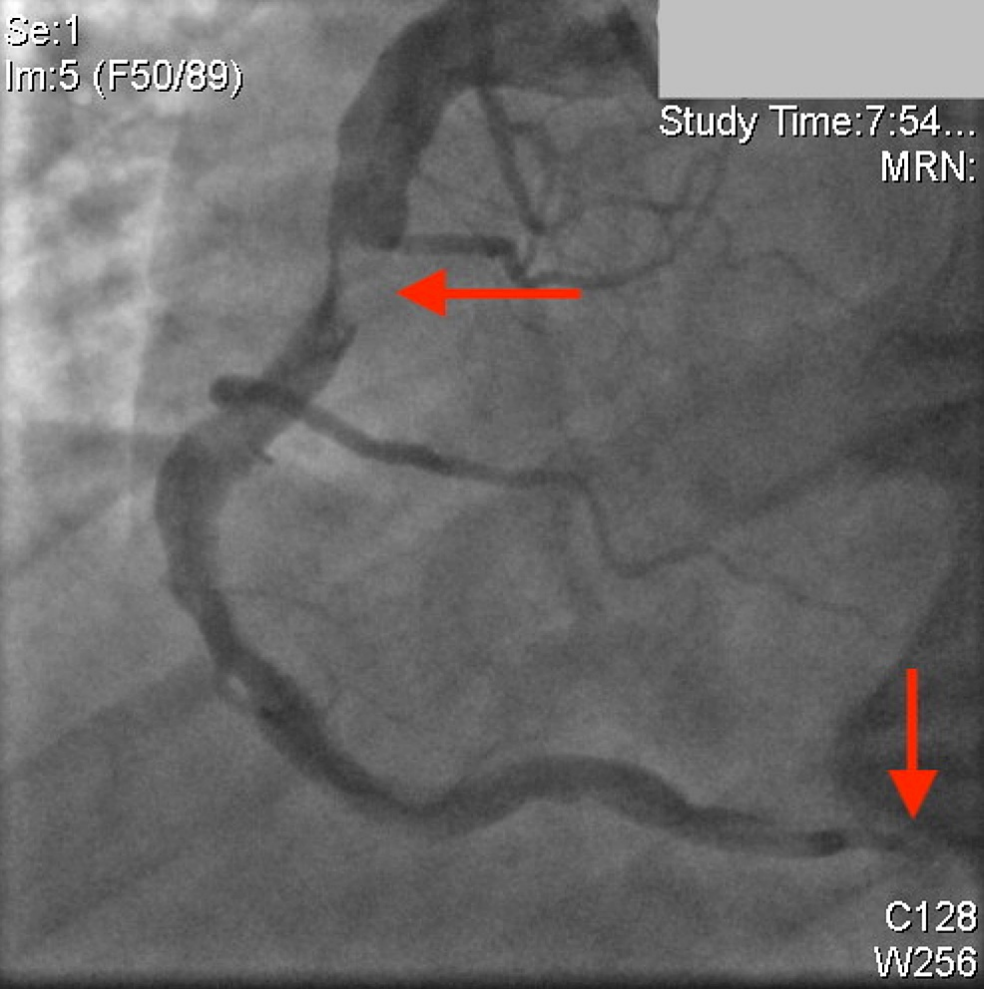
Right coronary artery

Coronary angiography demonstrated that the left anterior descending (LAD) artery, the ramus intermedius (RI), and the left circumflex artery (LCx) all had severe aneurysmal dilatation in the proximal part of the vessels. The remainder of the left-sided coronary vessels, including the left main artery (LMA), were large ectatic vessels with mild luminal irregularities (Figure 2). The RCA was a very large ectatic vessel, which was subtotally occluded with thrombus in the mid vessel with TIMI-1 flow distally (Figure 3). Additionally, there was another total occlusion in the right posterolateral branch proximally (Figure 3).

**Figure 4:**
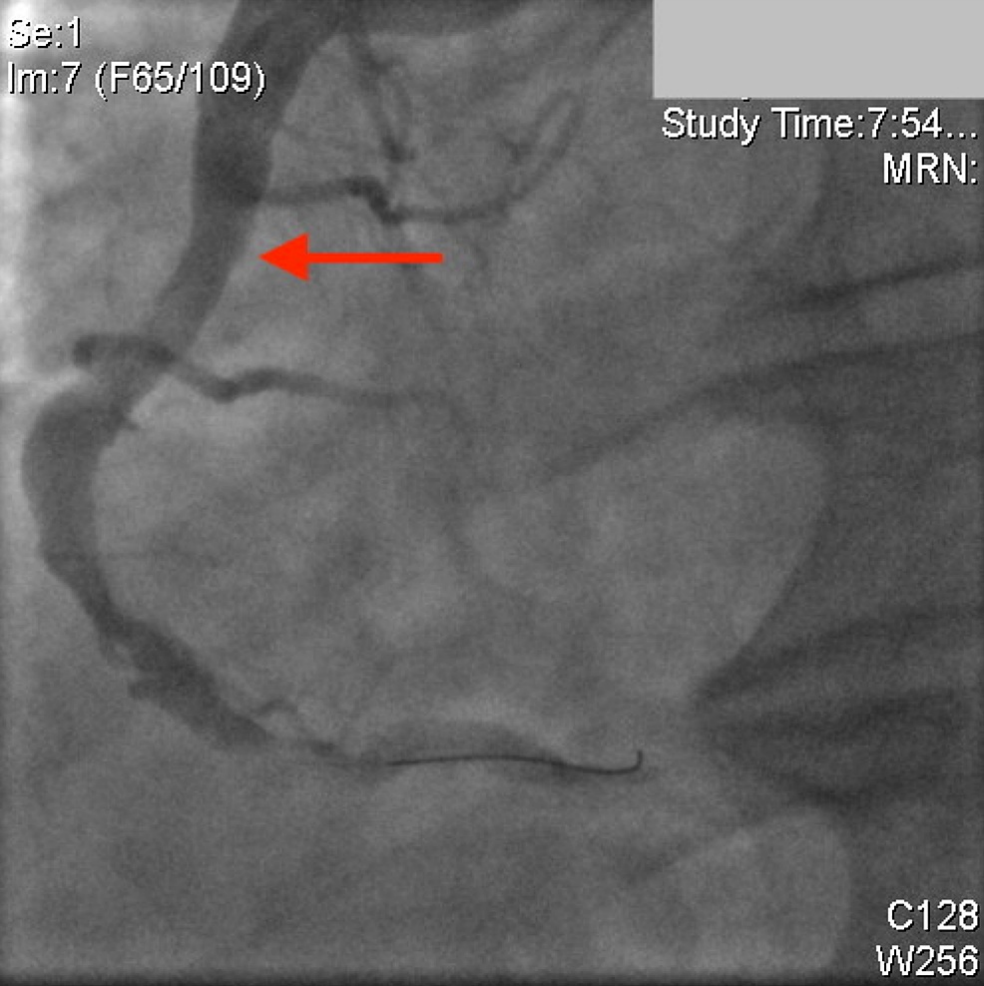
Right coronary artery following thrombectomy

Multiple slow aspiration runs were performed on the RCA, ultimately removing a large amount of thrombus. Repeat angiography showed complete resolution of the RCA thrombus and restoration of TIMI-3 flow into the distal vessels (Figure 4). There was no obvious lesion in the mid-RCA at the site of the thrombus, and it appeared similar to the rest of the vessel. 

In the right posterolateral branch, an aspiration catheter was advanced over a guidewire; however, it would not advance through the occlusion. Subsequently, aspiration was performed, which improved flow into the first and second branches of the posterolateral branch, but there was still evidence of a large thrombus burden. Thrombectomy was attempted at the dominant terminal posterolateral branch, but again the aspiration catheter would not advance.

**Figure 5:**
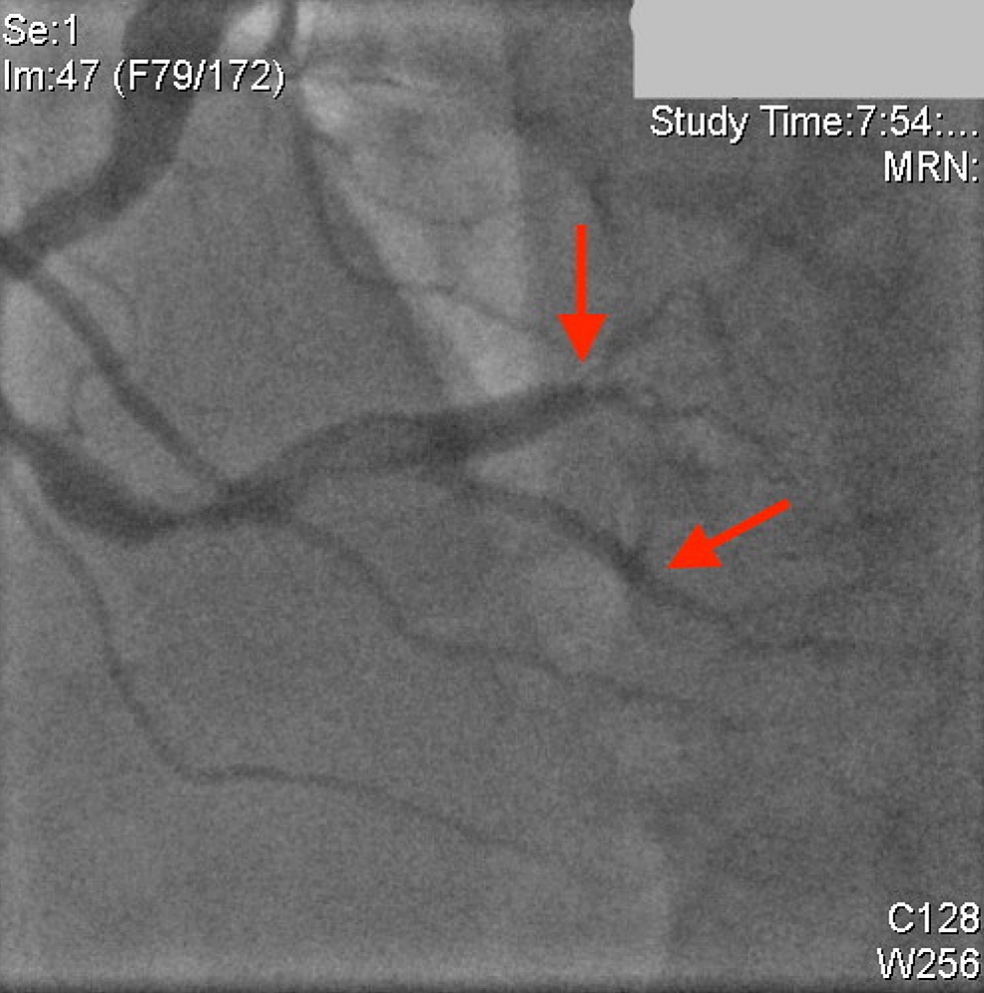
Right coronary artery with improved distal flow

This terminal posterolateral branch was then treated with multiple balloon angioplasty inflations throughout the vessel with very little, if any, improvement in flow in the terminal obtuse marginal. After a 2.75 mm balloon was inflated, there finally appeared to be a large vessel with a significant thrombus burden. Despite attempted thrombectomy, there was no significant improvement in the terminal posterolateral branch. However, the first posterolateral branch had significantly improved flow (Figure 5). 

**Figure 6:**
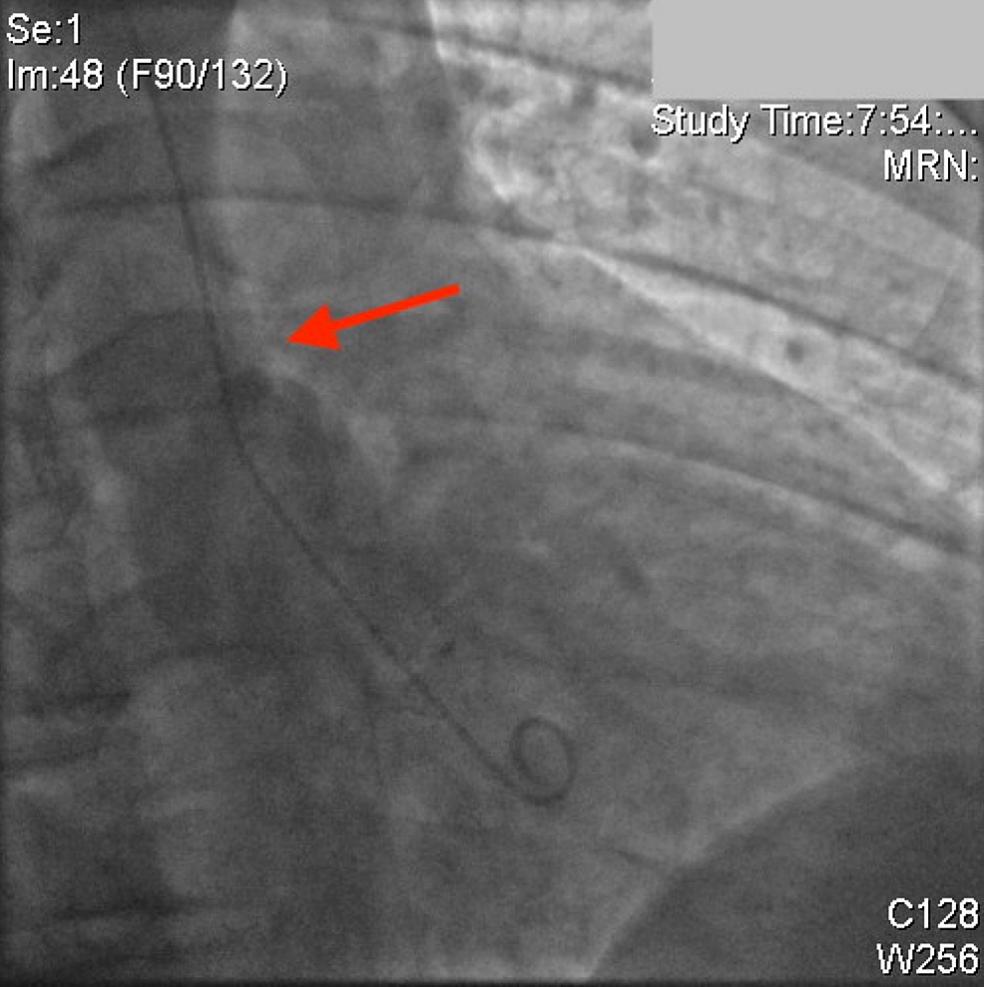
Normal aortography

It was determined that stenting was not an appropriate option because the appearance of the arteries resembled an inflammatory vasculitis disease process. Interestingly, there was no identified aortic dilation (Figure 6). After the thrombectomy and angioplasty, his ST segments did improve significantly though they did not completely resolve. Because of the ectatic nature of the vessels, the patient was treated with triple therapy including aspirin, clopidogrel, and rivaroxaban. At his two-month follow-up, he remained stable on triple therapy.

## Discussion

CAE is an unusual finding during angiography, and diffuse disease is even rarer. It is reported that the most commonly occurring sites for CAE are located in the RCA (68%), proximal LAD (60%), LCx (50%), and the left main being the rarest at only 0.1% [7]. CAE has mostly been associated with acquired etiologies, with atherosclerosis as the most predominant (Table 1). 

**Table 1:** Coronary artery ectasia etiologies Devabhaktuni et al., 2016 [7]

Etiology	Frequency
Atherosclerosis	50%
Kawasaki Disease	17%
Mycotic & Septic Emboli	11%
Connective Tissue Disease	<10%
Arteritis	<10%
Iatrogenic	Rare
Congenital	Rare

According to Doi et al., these coronary abnormalities are associated with an increased risk of a major adverse cardiac event (MACE) by 3.25 fold, cardiac death by 2.71 fold, and nonfatal myocardial Infarctions by 4.92 fold. They observed that ischemia occurred in nonobstructive coronary segments in greater than 70% of CAE patients who had a nonfatal myocardial Infarction. The suggested mechanism is thought to be a result of slow blood flow velocity through the dilated vessels, thereby increasing blood viscosity and coagulation. This led them to further study the outcomes of CAE patients receiving targeted therapeutic range (TTR) > 60% with warfarin, compared to those with TTR < 60%. They observed this group for 49 months and identified a reduced incidence of MACE by 33% in the group that had a TTR > 60%. While that is a substantial reduction, it should be noted that their population was limited to only eight participants receiving the TTR > 60% [8].

More recently, a retrospective study by Gunasekaran et al. examined the effects of DAPT (up to one year) versus anticoagulation (up to one year) versus either DAPT or anticoagulation in CAE patients with a mean follow-up period of 9.7 years. They analyzed the incidence of acute coronary syndrome (ACS) in 121 patients that received DAPT and 91 who did not receive DAPT and found that the DAPT group had significantly lower rates of ACS (17% vs 34%) on long term follow-up. Additionally, they analyzed 105 patients that received systemic anticoagulation and 91 that received no anticoagulation and similarly found that the anticoagulation group significantly lowered the incidence of ACS (29% vs 42%) on long-term follow-up [9].

Several studies have suggested that CAE has a high correlation with CAD. Liu et al. studied the progression of 99 CAE patients with a baseline coronary angiogram for up to 16 years when they received a comparative second coronary angiogram. They found that all CAE patients had existing atherosclerosis on their baseline angiogram, and atherosclerosis increased significantly on the follow-up angiogram. However, the ectasia showed minimal changes on the follow-up angiogram. They concluded that the prevention of worsening atherosclerosis may be the more clinically significant treatment modality compared to the treatment of ectasia [10].

## Conclusions

Our case of diffuse CAE in a young male, with a history of multiple family members dying from AMIs prior to the age of 40, redresses the question of complication prevention management. Due to the low prevalence of CAE in the population, there are limited studies addressing short and long term management. Medication management has not been standardized, and physicians have relied on their own clinical experiences. After a review of the literature that is available, we believe that it is likely appropriate to maximize treatment for atherosclerosis prevention in addition to choosing DAPT with or without systemic anticoagulation. If the disease continues to progress, despite maximizing medications, a heart transplant may be considered. There remains a need for completion of a large scale study to further address these concerns.

## References

[1] Willner NA, Ehrenberg S, Musallam A, Roguin A (2020). Coronary artery ectasia: prevalence, angiographic characteristics and clinical outcome. Open Heart.

[2] Gunes Y, Boztosun B, Yildiz A (2006). Clinical profile and outcome of coronary artery ectasia. Heart.

[3] Swaye PS, Fisher LD, Litwin P (1983). Aneurysmal coronary artery disease. Circulation.

[4] Zeina AR, Sharif D, Blinder J, Rosenschein U, Barmeir E (2007). Noninvasive assessment of coronary artery ectasia using multidetector computed tomography. Coron Artery Dis.

[5] Antoniadis P, Chatzizisis Y, Giannoglou G (2008). Pathogenetic mechanisms of coronary ectasia. Int J Cardiol.

[6] Alkhouli M, Gil I (2019). American College of Cardiology: Coronary ectasia. Coronary Ectasia. American College of Cardiology.

[7] Devabhaktuni S, Mercedes A, Diep J, Ahsan C (2016). Coronary artery ectasia-a review of current literature. Curr Cardiol Rev.

[8] Doi T, Kataoka Y, Noguchi T (2017). Coronary artery ectasia predicts future cardiac events in patients with acute myocardial infarction. Arterioscler Thromb Vasc Biol.

[9] Gunasekaran P, Stanojevic D, Drees T (2019). Prognostic significance, angiographic characteristics and impact of antithrombotic and anticoagulant therapy on outcomes in high versus low grade coronary artery ectasia: A long-term follow-up study. Catheter Cardiovasc Interv.

[10] Liu R, Zhao H, Gao X, Liang S (2021). Is coronary artery ectasia a progressive disease? A self-controlled retrospective cohort study. Front Cardiovasc Med.

